# CD8+ Cell Density Gradient across the Tumor Epithelium–Stromal Interface of Non-Muscle Invasive Papillary Urothelial Carcinoma Predicts Recurrence-Free Survival after BCG Immunotherapy

**DOI:** 10.3390/cancers15041205

**Published:** 2023-02-14

**Authors:** Julius Drachneris, Allan Rasmusson, Mindaugas Morkunas, Mantas Fabijonavicius, Albertas Cekauskas, Feliksas Jankevicius, Arvydas Laurinavicius

**Affiliations:** 1 Faculty of Medicine, Institute of Biomedical Sciences, Department of Pathology, Forensic Medicine and Pharmacology, Vilnius University, 01513 Vilnius, Lithuania; 2 National Center of Pathology, Affiliate of Vilnius University Hospital Santaros Klinikos, 08406 Vilnius, Lithuania; 3 Institute of Clinical Medicine, Faculty of Medicine, Clinic of Gastroenterology, Nephrourology and Surgery, Vilnius University, 01513 Vilnius, Lithuania; 4 Center of Urology, Vilnius University Hospital Santaros Klinikos, 08410 Vilnius, Lithuania

**Keywords:** computational pathology, digital pathology, artificial intelligence, tumor-infiltrating lymphocytes, anti-tumor immune response, tumor microenvironment, predictive model, immunotherapy

## Abstract

**Simple Summary:**

Bacille Calmette–Guerin (BCG) immunotherapy of non-muscle invasive papillary urothelial carcinoma fails in over 30% of cases. In our study, we explore the significance of tumor-infiltrating cytotoxic lymphocytes, assessed by digital analysis and computational methods measuring the cell gradient density profiles across the tumor epithelium–stroma interface, to predict recurrence-free survival in these patients. We analyzed CD8+ cell distribution profiles in the tumor tissue using previously published methods of gradient assessment (center of mass and immunodrop) along with patients’ clinical and pathology data. We found that both CD8+ cell gradient indicators were statistically significant prognosticators of recurrence-free survival, and together with clinical and pathological data might be used for improved patient risk stratification. In this context, we propose a prototypic risk assessment system incorporating pathology, patients’ history, and CD8+ cell gradient features.

**Abstract:**

Background: Bacille Calmette–Guerin (BCG) immunotherapy is the first-line treatment in patients with high-risk non-muscle invasive papillary urothelial carcinoma (NMIPUC), the most common type of bladder cancer. The therapy outcomes are variable and may depend on the immune response within the tumor microenvironment. In our study, we explored the prognostic value of CD8+ cell density gradient indicators across the tumor epithelium–stroma interface of NMIPUC. Methods: Clinical and pathologic data were retrospectively collected from 157 NMIPUC patients treated with BCG immunotherapy after transurethral resection. Whole-slide digital image analysis of CD8 immunohistochemistry slides was used for tissue segmentation, CD8+ cell quantification, and the assessment of CD8+ cell densities within the epithelium–stroma interface. Subsequently, the gradient indicators (center of mass and immunodrop) were computed to represent the density gradient across the interface. Results: By univariable analysis of the clinicopathologic factors, including the history of previous NMIPUC, poor tumor differentiation, and pT1 stage, were associated with shorter RFS (*p* < 0.05). In CD8+ analyses, only the gradient indicators but not the absolute CD8+ densities were predictive for RFS (*p* < 0.05). The best-performing cross-validated model included previous episodes of NMIPUC (HR = 4.4492, *p* = 0.0063), poor differentiation (HR = 2.3672, *p* = 0.0457), and immunodrop (HR = 5.5072, *p* = 0.0455). Conclusions: We found that gradient indicators of CD8+ cell densities across the tumor epithelium–stroma interface, along with routine clinical and pathology data, improve the prediction of RFS in NMIPUC.

## 1. Introduction

Bladder cancer is the tenth most common cancer diagnosed in the world [1], with around three-fourths of the cases being non-muscle-invasive bladder cancer (NMIBC) [2]. Several risk assessment systems have been developed based on traditional tumor properties (grade, stage, size, multifocality, presence of carcinoma in situ, and previous history of recurrence) to support therapy decisions for NMIBC patients [3,4,5,6]. For high- and intermediate-risk patients, the main option of adjuvant treatment is intravesical Bacille Calmette–Guérin (BCG) immunotherapy, which has been demonstrated to reduce relapse, progression, and death rates in NMIBC patients [2]. Nevertheless, over 30% of patients experience tumor recurrence after BCG immunotherapy; therefore, better predictive and clinical-decision support tools are in demand [7].

Cancer immunotherapy advances over the last few years are demanding better assessment of the tumor microenvironment [8]. As suggested by Song et al. [9], biological features (high mutational rate, mismatch repair, and DNA damage response deficiencies) of bladder cancer, along with current treatment strategies, make this tumor a good model to understand anti-tumor immune response mechanisms. This led to studies focusing on subsets of cells in the bladder cancer microenvironment, revealing their impact on patient outcomes. However, most of these studies were focused on muscle-invasive bladder cancer (MIBC) [10]. In the subset of NMIBC treated with BCG, evidence for the prognostic significance of specific cell subpopulations has been reported for eosinophils [11], tumor-associated macrophages (TAM) [12,13,14], dendritic cells [12], tumor-infiltrating lymphocytes (TILs) [13], M1/M2 TAM subsets [15,16,17,18], and TIL (T cells subsets, B cells) subpopulations [11,19,20,21,22].

TILs were most extensively investigated, revealing potential clinical utility and leading to international initiatives to standardize TIL assessment in a variety of tumors [23,24]. Major progress has been made with novel opportunities brought by digital image analysis (DIA); this has enabled the high-capacity assessment of TILs, also exploring spatial aspects and multiple associations of cell subtypes [25]. As an example, the “Immunoscore” system, proposed by Galon et al. [26] for colorectal cancer, estimates not only the absolute densities of TILs but also their distributions in the tumor compartments. This method was later validated in a large multicentric study [27] and adapted to other tumor types [28]. Recently, Bieri et al. presented a “modified Immunoscore” (mIS) from DIA of tissue microarrays and confirmed their mIS to be an independent prognosticator of clinical outcomes in patients with muscle-invasive bladder cancer [29]. However, in a subsequent study of NMIBC patients, this indicator was only of prognostic significance in the high-risk patient subgroup [22].

Recently, Rasmusson [30] proposed an automated tumor–stroma interface zone (IZ) sampling method, with the subsequent computation of Immunogradient indicators, rather than measuring absolute TIL densities in tumor compartments. This method enables the selective and extensive sampling of the tumor–host interaction area with the quantification of the TIL density gradient across it. These indicators were tested as independent prognostic computational biomarkers in colorectal and breast cancer patients [30,31,32,33]. Their performance in the context of immunotherapy has not been investigated.

Non-muscle-invasive papillary urothelial carcinoma (NMIPUC) comprises the vast majority of NMIBC and is defined by papillary structure formation [34]. This specific tumor architecture, along with a lack of conventional invasive growth patterns in the majority of cases, may require a particular approach to assess TILs. Therefore, in this study, we explored the prognostic significance of CD8+ cell density profiles quantified as immunogradient indicators in the delicate architectural context of NMIPUC. We found that a relative decrease in CD8+ cell densities across the narrow range (40 micrometers) of the epithelial–stroma interface was an independent prognostic marker of shorter RFS in the patients after BCG immunotherapy.

## 2. Materials and Methods

Clinical and pathological data of urinary bladder cancer patients treated with BCG intravesical immunotherapy in Vilnius University Hospital Santaros Klinikos (Vilnius, Lithuania) between 2008 and 2020 (230 in total) were collected. A total of 165 patients with NMIPUC, with a full 6-week BCG induction course and available TUR resection material, were included. After performing tissue sections and IHC (see below), 8 patients with insufficient material were removed from the study. The demographic, clinical, and pathological data of 157 patients are summarized in Table 1. Recurrence-free survival (RFS) time was calculated from the day of the first BCG induction to the date of the first documented tumor recurrence. RFS times were censored after 5 years of follow-up because later recurrences might not represent the true recurrence of a previously diagnosed tumor but the development of a new primary cancer lesion [35].

Archival slides were reviewed by a pathologist (J.D.) who selected the most informative (containing the highest grade and invasive tumor area if present) formalin-fixed paraffin-embedded (FFPE) tissue block. Next, 3 µm tissue sections were stained for CD8 (Dako, clone C8/144B, dilution 1:100, Denmark, using the ultraView Universal DAB Detection kit, Ventana Medical Systems, Oro Valley, AZ, USA) on a Roche Ventana BenchMark ULTRA automated stainer (Ventana Medical Systems, USA).

**Figure 1:**
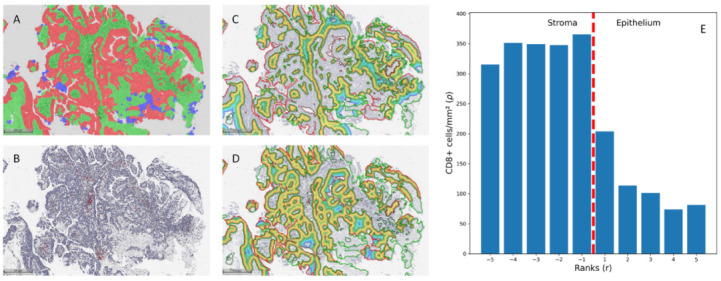
(**A**). Tissue classification results. (**B**). Cell classification results. (**C**,**D**). Infiltration analysis was performed on stromal and epithelial compartments. The 500 µm measures were added for reference. (**E**). CD8+ cell density distribution by ranks in an example case.

All CD8 IHC slides were digitized at 20× magnification (0.5 µm per pixel) using an Aperio^®^ AT2 DX scanner (Leica Aperio Technologies, Vista, CA, USA). The HALO^®^ AI (Indica Labs, USA) Densenet v2 classifier was trained using manual annotation provided by the pathologist (J.D.), including the classes ‘stroma’, ‘epithelium’, and ‘artifacts’ (Figure 1A). The latter was added to exclude areas of coagulation that might affect spatial analysis and necrotic areas, hemorrhage, or calcifications. The images were reviewed by a pathologist (J.D.), and epithelial region boundaries shorter than 1000 µm were removed to reduce noise caused by small, incorrectly classified foci. The tissue classification was followed by CD8+ cell segmentation (Figure 1B) and CD8+ cell distribution in the stroma–epithelium interface zone (Figure 1C,D) using HALO^®^ Multiplex IHC and Spatial Analysis modules (Indica Labs, Albuquerque, NM, USA), respectively. The spatial analysis was performed on a 150 µm zone divided into 10 µm width bands in both the epithelial and stromal sides, assigning ranks according to the distance from the epithelium–stroma interface (e.g., rank 1—covering area ranging from the interface (0 distance) to 10 µm; rank 2—from 10 µm to 20 µm; rank 3—from 20 µm to 30 µm, etc.) with a negative or positive rank value assigned to the stromal or to epithelial aspect, respectively (Figure 1E).

The immunodrop (ID) and center of mass (CM) immunogradient indicators [30] were adapted to the results of the infiltration analysis. ID was calculated as the ratio of CD8+ cell density in a corresponding pair of stromal and epithelial bands (e.g., ID (5) is the ratio between CD8 cell density in the stromal band with rank −5 and the epithelial band with rank 5).

$$\mathrm{ID}=\frac{\rho_{-r}}{\rho_{r}},$$

where *ρ* represents CD8+ cell density in the band, and *r* represents the rank of the band number. CM was calculated as the ratio between the sum of the products of the indices and densities of bands in the IZ and the total sum of CD8+ cell densities in the IZ.

$$\mathrm{CM}=\frac{\sum_{r_{i}}r_{i}\times \rho_{i}}{\sum_{r_{i}}\rho_{i}}$$

Additionally, absolute CD8+ cell densities were calculated in the stromal and epithelial compartments and the overall IZ area. To search for the optimal width of the IZ, absolute densities of CD8+ cells and CM gradient indicators were assessed at various IZ widths and ranks, ranging from 20 to 300 µm.

The dataset was randomly split into a training set (117 patients) and a hold-out test set (40 patients) with a similar proportion of patients with tumor recurrence in both sets. The univariable Cox model was used to evaluate the performance of individual features and to select features for multivariable Cox regression. At this point, we had multiple variants of ID, CM, and absolute densities in tumor compartments. The variant with the lowest *p*-value was selected. For multivariable analysis, we selected factors from univariable analysis with *p*-value < 0.05, constructing all possible combinations of these variables for modeling. Multivariable Cox regression models using these constellations of variables were then fitted on the training set to select models consisting of independent variables (with *p*-values of HR for all covariates being <0.05). On these selected models, we performed 5-fold cross-validation using the mean Harrell’s C-index on the validation set as a performance indicator. We then selected the best-performing model and tested it on a hold-out test set. The Kaplan–Meier survival estimator was used to investigate the survival of patients stratified according to factors with statistically significant association with recurrence hazard in the univariate Cox analysis, and the log rank test was used for the pairwise comparison of patient survival in groups. For prognosticators with continuous data (ID and CM), we have stratified patients into three equal groups (low, medium, and high). Statistical data analysis was performed using Python libraries (Pandas version 1.3.4, Scikit-learn version 1.0.2 and Lifelines version 0.27.0).

## 3. Results

### 3.1. Univariable Cox Regression Analysis

The tumor stage and G3 tumor grade were significantly associated with increased recurrence risk (data summarized in Table 2). Other traditional stratification prognosticators, such as tumor size, concurrent CIS, tumor multifocality and demographic data (age and gender), did not show statistically significant association.

Both CD8+ cell density gradient indicators (CM and ID) were significantly associated with patient outcomes, while absolute CD8+ cell densities in the stromal, epithelial, and overall IZ compartments failed to show significant association with RFS. The best-performing variation of ID included a ratio of ranks 10–20 µm on stromal and epithelial sides, suggesting that the changes closest to the epithelial–stromal interface are most indicative of patient outcomes. The worse performance of the ID variant using band ranks next to the epithelial–stromal interface (0–10 µm) might be associated with minor inconsistencies in tissue classifier performance. Similarly, the best-performing CM measure was obtained from an IZ covering the 0–20 µm interval from the interface on both epithelial and stromal aspects (ranks −2 to 2).

Of the medical history data, the positive reTUR but not the recurrent tumor was associated with significantly increased recurrence hazard. Interestingly, the combination of these two factors formed an even stronger predictor of BCG failure (the HR of patients having positive reTUR and/or recurrent tumor was 5.4702 in comparison with only having positive reTUR patients HR of 3.6726).

### 3.2. Multivariable Cox Regression Analysis

A total of 11 multivariable Cox regression models could be obtained from the univariate prognostic features (Table 3). The best-performing model from the 5-fold cross-validation included ID, G3 tumor grade, and positive reTUR or recurrent tumor (Table 4). Other models showed a slightly higher Akaike information criterion (AIC) and lower mean C-index in the validation splits. Some of these models included CM but without the co-occurrence of ID in the same model. The strongest models included the covariates of any medical history parameter with positive anamnesis of a tumor (positive reTUR and/or recurrent tumor), thus showing the high predictive value of this feature.

The best-performing model from the 5-fold cross-validation included ID, G3 tumor grade, and positive reTUR or recurrent tumor (Table 3) (log-likelihood ratio = 22.76, *p* < 0.005). Of note, this model included one anamnestic factor, one histological factor, and one tumor microenvironment factor. The C-index for the test set was 0.7429, slightly lower than the training set C-index of 0.7579, which excludes the possibility of overfitting this model.

### 3.3. Kaplan–Meier Survival Analysis

**Figure 2:**
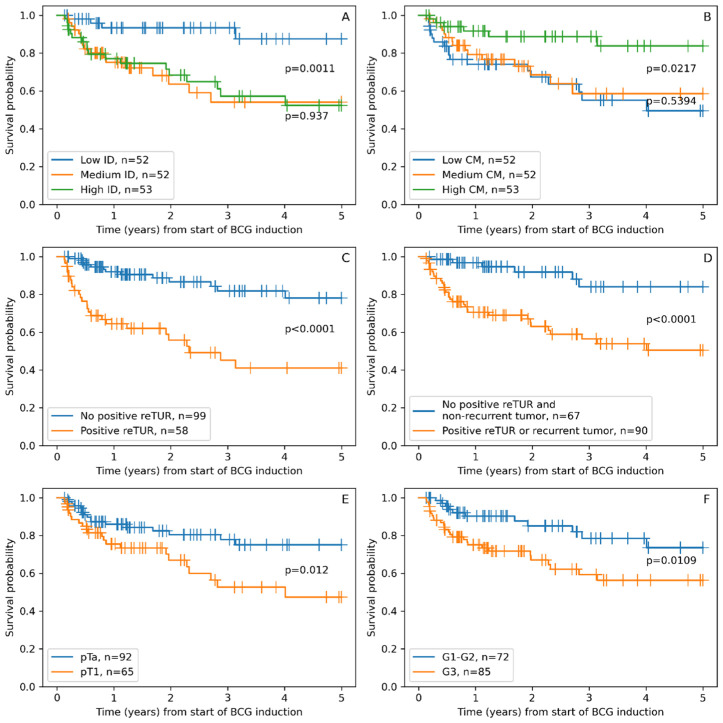
Kaplan–Maier RFS plots stratified according to: (**A**). Immunodrop (ID), (**B**). Center of mass (CM), (**C**). Presence of tumor in reTUR, (**D**). Presence of tumor in repeated transurethral resection (reTUR) or recurrent tumor, (**E**). Tumor stage (pTa vs. pT1), (**F**). Tumor grade (G3 vs. G1–G2).

Non-parametric survival analyses once again supported our finding from univariable Cox regression analysis, with all six features separating patient groups with statistically significant differences in RFS (Figure 2). The low ID group of patients showed significantly longer RFS, similar to the high CM group, which was expected due to the strong inverse correlation of these indicators. The traditional pathologic factors of tumor stage and grade also separated groups with significantly different RFS. The presence of tumors in reTUR was associated with a significantly shorter RS. However, the combined factor of recurrent tumors and/or the presence of tumors in reTUR extracted a larger group of patients with shorter RFS, resulting in a somewhat better balanced risk stratification.

**Figure 3:**
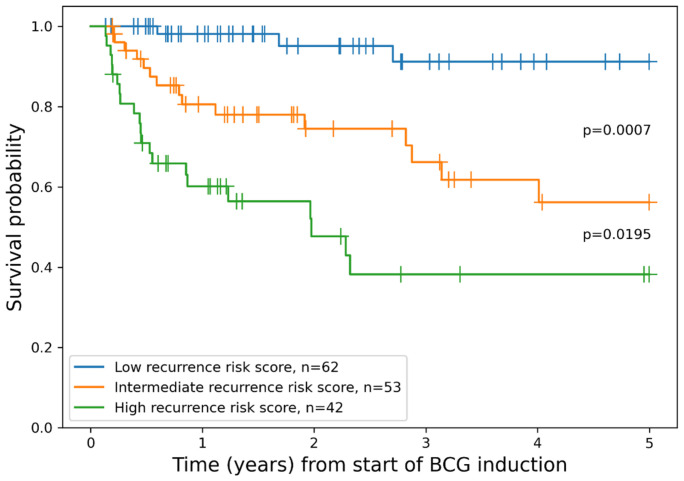
Kaplan–Meier plot of RFS grouped by combined model for patient risk assessment.

Additionally, we constructed a combined risk assessment score based on three independent factors included in the best-performing Cox regression model. A score of 1 was added for each G3 tumor grade, positive reTUR or recurrent tumor, and medium or high ID. For the final stratification, patients having 0 or 1 point were assigned to the “low recurrence risk score” group, thus forming a more balanced patient distribution between groups; patients with 2 points were assigned to the “intermediate recurrence risk score” group; and patients with 3 points to the “high recurrence risk score” group. This scoring system enabled statistically significant risk stratification in regard to RFS (Figure 3). 

## 4. Discussion

Our study reveals that CD8+ cell density gradient indicators, ID and CM, were significantly associated with the RFS of patients treated with BCG immunotherapy for NMIPUC, highlighting the importance of the spatial distribution of CD8+ cells across the tumor interface. Similar work published recently by Bieri et al. [22,29] explored the prognostic significance of TILs in bladder cancer by introducing the mIS concept for the assessment of TILs in the tumor tissue. In both of their studies, mIS enabled significant risk stratification only in subsets of patients (progression-free survival and cancer-specific survival stratification in MIBC after cystectomy—the AJCC stage IIIa group, RFS stratification in NMIPUC treated with BCG—EORTC high-risk group). In contrast, the CD8+ Immunogradient indicators provided significant stratification in the entire cohort of our patients.

An important advantage of our method is that it generates the CD8+ cell density data from the epithelium–stroma interface with high selectivity and capacity while also maintaining the spatial context of the tumor–host interaction area. In the univariate analyses, best-performing variants of both CM and ID were generated from the IZ within the range of 20 µm into both the stromal and epithelial aspects. DIA performed a precise selection of areas of interest and, paired with the high-throughput nature of the method in WSI, enabled an optimized solution to assess tissue immune response in this tumor with a peculiar papillary microarchitecture. In contrast to other tumor types, where immunogradient indicators and Immunoscore were found to be prognostic, NMIPUC, in most cases, does not have a wide invasive border. Instead, tumor–host interaction takes place in a very thin, elongated, papillary tumor structure, requiring a more delicate approach. Our study shows that this can be achieved with AI-based pixel-level tissue classification with subsequent computational immunohistochemistry assessment.

Another important observation emerging from our study is that CD8+ cell density gradient indicators were significant prognosticators of RFS, while none of the absolute CD8+ cell densities in any tumor tissue compartment showed a significant impact. This further supports the importance of spatial analytics to study tumor microenvironments rather than relying on the quantification of cell densities in tumor tissue compartments. Whereas all patients in our cohort received BCG immunotherapy, our computational models enable the assessment of RFS probability and, with an appropriate study design, can be tested as predictive biomarkers for immunotherapy modalities.

Based on three independent prognostic factors in our best-performing multivariable Cox regression model after cross-validation procedures, we constructed a scoring system which, importantly, combines clinical, pathology, and immune response features. The model enables RFS probability assessment by assigning the patients after BCG immunotherapy into three risk categories (Figure 3). To compare our model to the current routine risk assessment strategy, we simulated the performance of the EORTC risk stratification algorithm (REF) in our patient cohort. The EORTC risk groups provided statistically significant differences only between intermediate and very high-risk groups (*p* = 0.0448), while other pairwise differences did not reach statistical significance (intermediate vs. high *p* = 0.1698, high vs. very high *p* = 0.1073). This “underperformance” of the EORTC scheme might be explained by some shift toward more aggressive tumors in our patients, eligible for BCG immunotherapy. Additionally, the impact of BCG on RFS and/or the limited sample size of our study remains to be considered. Nevertheless, we found that our scoring scheme was best-performing in our patient cohort and remains to be tested for its potential in clinical decision making.

The evidence on the use of early radical cystectomy for high-risk non-muscle-invasive bladder cancer that can be performed upfront or in a delayed setting after BCG failure remains controversial [36]. However, many recent reports have shown that in patients with BCG-unresponsive HGT1 disease, cancer-specific and overall survival were lower after delayed (>2 years) versus early radical cystectomy [37,38,39]. However, the retrospective series suggested that in patients with T1G3 tumors, there was only a small difference in recurrence rate between the BCG-treated and the non-BCG-treated group (70 vs. 75%) [40]. Nearly 40% of patients in our study harbored HGT1 disease and, therefore, were less likely to respond to BCG therapy than high-grade Ta tumors. In this context, any improvement of pretreatment prognostic stratification may have very high clinical importance, improving oncological outcomes in significant numbers of patients or/and sparing them from excess radical cystectomy.

Our study has some limitations. Small inconsistencies in our tissue classifier performance caused some misclassified epithelial areas in the stroma which required some manual data curation. It was performed in a standardized manner, nevertheless, making the DIA not entirely automated. Another issue in urothelial tumors is the cytological similarity of the malignant and nonmalignant urothelium, which is why the interface zone in our study includes any urothelium. Therefore, all tissue has been classified as ‘stroma’ or ‘epithelium’. However, to reduce the impact of data derived from normal mucosa, we have selected tissue samples for the study with a predominance of tumor epithelium over normal urothelium.

## 5. Conclusions

Our study reveals an independent informative value of CD8+ cell density gradient across the epithelium–stroma interface to predict RFS in patients with NMIPUC treated with BCG immunotherapy. Importantly, absolute CD8+ cell densities in the tumor epithelia or stroma compartments did not reveal any prognostic impact. This further supports the advantage of immunogradient indicators to assess patterns of infiltrating immune cell distribution in the tumor microenvironment. Combining CD8+ immunogradient with the patient’s history of reTUR and histological grade of the tumor, we propose a risk assessment score to predict RFS in patients with NMIPUC after BCG immunotherapy.

## Figures and Tables

**Table 1:** Summary of clinical and pathologic data.

Characteristic	Value (%)
Patients	157 (100%)
Age, years	
Median (range)	69.8 (33–89)
Gender	
Male	128 (81.5%)
Female	29 (18.4%)
RFS time, months	
Median (range)	16.6 (1–174)
Recurrences (BCG failures)	39 (24.8%)
Tumor grade	
G1	5 (3.1%)
G2	67 (42.7%)
G3	85 (54.1%)
pT stage	
Ta	95 (60.5%)
T1	62 (39.5%)
Carcinoma in situ association	8 (5.1%)
Positive reTUR *	58 (36.9%)
Recurrent tumor **	47 (29.9%)
Positive reTUR * or recurrent tumor	90 (57.2%)
Multiple tumors	78 (49.7%)
Tumor size > 30 mm	43 (27.4%)
EORTC risk group	
Intermediate	76 (48.4%)
High	72 (45.9%)
Very High	5 (3.1%)

* NMIPUC identified on repeated transurethral resection; ** not the primary NMIPUC identified on the first transurethral resection.

**Table 2:** Univariable Cox regression results.

Feature	*p*-Value	HR
Male gender	0.6229	0.7845
Age	0.4051	1.0000
Immunodrop	0.0031	12.2830
Center of mass	0.0082	0.0660
CD8 density epithelial	0.1140	0.9971
CD8 density stromal	0.2718	0.9993
CD8 density overall	0.1659	0.9979
pT1 stage	0.0126	2.6092
G1	0.9959	0.0000
G2	0.0757	0.4773
G3	0.0159	2.7387
Concurrent CIS	0.4793	1.5417
Tumor size > 30 mm	0.5781	0.7686
Multiple tumors	0.4050	1.3858
Positive reTUR *	0.0009	3.6726
Recurrent tumor **	0.3955	1.3945
Positive reTUR * or recurrent tumor	0.0016	5.4702
EORTC Intermediate risk	0.0655	0.4765
EORTC High risk	0.0766	1.9712
EORTC Very high risk	0.2071	2.5514

* NMIPUC identified on repeated transurethral resection; ** not the primary NMIPUC identified on the first transurethral resection.

**Table 3:** Performance of multivariable Cox regression models (CM—center of mass, ID—immunodrop).

Model Covariates	Mean Validation Set C-Index	AIC
Positive reTUR * or recurrent tumor ** + G3 + ID	0.7837	173.3428
Positive reTUR * or recurrent tumor ** + G3	0.7397	174.6718
Positive reTUR * or recurrent tumor ** + ID	0.7370	174.7917
Positive reTUR * or recurrent tumor ** + pT1 + ID	0.7388	172.5348
Positive reTUR * or recurrent tumor ** + pT1	0.7355	174.4835
Positive reTUR * or recurrent tumor ** + CM	0.7308	174.9942
G3 + ID	0.7028	179.8105
G3 + CM	0.7028	179.8105
Positive reTUR + ID	0.6613	178.3879
pT1 + CM	0.6551	180.2151
pT1 + ID	0.6438	178.4539

* NMIPUC identified on repeated transurethral resection; ** not the primary NMIPUC identified on the first transurethral resection.

**Table 4:** Best-performing multivariable Cox regression model.

Covariate	*p*-Value	HR
Positive reTUR * or recurrent tumor **	0.0063	4.4492
G3	0.0457	2.3672
ID	0.0455	5.5072

* NMIPUC identified on repeated transurethral resection; ** not the primary NMIPUC identified on the first transurethral resection.

## Data Availability

Data presented in this study can be obtained from the author on request. These data are not available to the public due to permit restrictions.
